# Lysophosphatidylcholine acts in the constitutive immune defence against American foulbrood in adult honeybees

**DOI:** 10.1038/srep30699

**Published:** 2016-08-02

**Authors:** Ulrike Riessberger-Gallé, Javier Hernández-López, Gerald Rechberger, Karl Crailsheim, Wolfgang Schuehly

**Affiliations:** 1 Institute of Zoology, Universitätsplatz 2, University of Graz, 8010 Graz, Austria; 2 Institute of Molecular Biosciences, University of Graz, NAWI Graz, Humboldtstraße 50/II, 8010 Graz, Austria; 3 OMICS Centre Graz, BioTechMed-Graz, 8010 Graz, Austria; 4 Institute of Pharmaceutical Sciences, Pharmacognosy, Universitätsplatz 4, University of Graz, 8010 Graz, Austria

## Abstract

Honeybee (*Apis mellifera*) imagines are resistant to the Gram-positive bacterium *Paenibacillus larvae* (*P. larvae*), causative agent of American foulbrood (AFB), whereas honeybee larvae show susceptibility against this pathogen only during the first 48 h of their life. It is known that midgut homogenate of adult honeybees as well as a homogenate of aged larvae exhibit strong anti-*P. larvae* activity. A bioactivity-guided LC-HRMS analysis of midgut homogenate resulted in the identification of 1-oleoyl-*sn*-glycero-3-phosphocholine (LPC) pointing to a yet unknown immune defence in adult honeybees against *P. larvae*. Antimicrobial activity of LPC was also demonstrated against *Melissococcus plutonius*, causative agent of European Foulbrood. To demonstrate an AFB-preventive effect of LPC in larvae, artificially reared larvae were supplemented with LPC to evaluate its toxicity and to assess whether, after infection with *P. larvae* spores, LPC supplementation prevents AFB infection. 10 μg LPC per larva applied for 3 d significantly lowered mortality due to AFB in comparison to controls. A potential delivery route of LPC to the larvae in a colony via nurse bees was assessed through a tracking experiment using fluorescent-labelled LPC. This yet undescribed and non-proteinous defense of honeybees against *P. larvae* may offer new perspectives for a treatment of AFB without the utilization of classic antibiotics.

Defence strategies in insects against pathogens rely on individual innate mechanisms, such as the action of antimicrobial peptides, e.g., apidaecins in honeybees^1^, or specifically induced immune responses that infer an immunological memory, including transgenerational immune priming effects^2^,^3^,^4^. Further, a suppression of pathogenic bacteria by the intestinal probiotic lactic acid bacterial community can be observed in *Apis mellifera*^5^,^6^. In addition to individual immune strategies, social immunity, which is a defense form based on behavioral patterns and whose efficiency increases with the sociability of the respective eusocial insect, plays a major role, particularly in honeybees^7^,^8^,^9^,^10^.

American foulbrood (AFB) is a devastating bee disease that is caused by the Gram-positive spore-forming bacterium *Paenibacillus larvae* (*P. larvae*), for which several genotypes including ERIC I and ERIC II have been identified, which show different pathogenic behaviour^11^,^12^. The spores of *P. larvae* germinate in the larval midgut, from which – after proliferation – they invade through the midgut epithelium into the hemocoel^13^. Infected larvae die from septicaemia and turn into a ropy mass before pupation occurs. Subsequently, spores of *P. larvae* are formed and transferred by adult honeybees that clean the cells and healthy larvae are infected by nursing. This initiates a cycle of re-infection that generally results in the death of the bee colony. Already in 1937, Tarr^14^ demonstrated that while the vegetative stages of *P. larvae* are not infectious, the spores – the only infectious form – remain infectious for long periods^15^. The dose-dependent susceptibility of bee larvae to *P. larvae* spores from different strains or wild isolates has been demonstrated for both larvae reared by nurse bees^16^,^17^ and artificially reared larvae^18^.

For the treatment of AFB, the use of antibiotics is allowed in the US and Canada despite general concerns about negative consequences such as the development of resistance and antibiotic residues in honey^19^. In Europe, where the use of antibiotics in beekeeping is not permitted, beekeepers are required to undertake drastic measures such as immediate quarantining and burning of hives when AFB infection becomes clinically.

Honeybee larvae are susceptible to *P. larvae* spores only during the first instar stage and become resistant against the infection after the first approximately 48 h of life. This age-dependent decrease in susceptibility, which according to some authors goes along with tissue differentiation in the peritrophic matrix^13^, has intrigued many researchers and has formed the starting point of our research. It is known that the infection process is accompanied by histopathological changes in the larval midgut^20^. It was further shown that total degradation of the peritrophic matrix is necessary before *P. larvae* can affect epithelial cells^21^, however, we conjectured these mechanisms to be complemented by the involvement of a chemical substance, tentatively also in the case of European foulbrood (EFB), which is a potentially lethal bee brood disease that is also caused by a Gram-positive bacterium, *Melissococcus plutonius* (*M. plutonius*)^22^.

We previously reported on the detection of a non-induced heat-stable substance present in the midgut of adult bees that shows strong antibacterial activity against the vegetative stage of different *P. larvae* isolates occurring in Central Europe^23^,^24^,^25^. We demonstrated that about one tenth of a preparation of a single homogenized midgut of an adult honeybee – equaling approximately 50 μg of dry weight tissue – inhibited the growth of vegetative forms of *P. larvae* in an overnight culturing experiment. In histological examinations, antimicrobial activity was demonstrated in the peritrophic membrane and isolated midgut tissue^26^. Moreover, tests of homogenized larvae (day 3 and older) revealed a positive correlation between larval age and antibacterial activity^25^. This antimicrobial activity was retained even after heat treatment, ethanolic precipitation, and the application of proteases, prompting us to focus our attention on a non-proteinogenic source of *P. larvae* resistance.

In this paper, we report on the identification of 1-*O*-oleoyl-*sn*-glycero-3-phosphocholine (l-α-lysophosphatidylcholine, lysolecithin, LPC) isolated from honeybee midguts and conferring antimicrobial activity against the causative agents of bacterial brood diseases. We demonstrate the growth-inhibitory effect of LPC towards *P. larvae* both *in vitro* and *in vivo* and provide evidence that argues the anti-AFB beneficial effect of LPC supplementation of larval food (proof of principle). Additionally, we describe the anti-*P. larvae* and anti-*M. plutonius* activity of several known lipidic compounds that share chemical features of LPC, and provide a model to demonstrate through fluorescence-tracking the achievement of a relevant LPC concentration in the larvae of a colony. These findings may allow the development of an AFB treatment that circumvents the application of antibiotics.

## Results

### Identification of LPC as an antibacterial compound in midgut tissue

To elucidate the nature of the anti-*P. larvae* activity in the adult honeybee’s midgut, we analysed and characterized the bioactive component in an aqueous ethanolic extract of honeybee midgut. In a bioactivity-guided fractionation procedure involving solid phase extraction (SPE) and semi-preparative HPLC, the anti-*P. larvae* activity was localized in the late eluting fractions at 90–95 and 95–100 min, respectively (see electronic supplementary material, Fig. S1). The elution profile indicated a highly lipophilic nature of the antimicrobial substance, and ruled out that lactic acid, produced by a potentially occurring lactic acid bacterial community in the midgut, was responsible for the antibacterial effect. Exposure of the active fractions to either proteases or heat (120 °C) did not substantially alter the anti-*P. larvae* activity^23^. Filtering using size exclusion centrifugal filter units (cut-offs 3,000 and 30,000 Da, respectively) left the biological activity in both cases in the supernatant pointing to the possibility of a lipidic compound in micellar state. A consecutive sampling of the 95–105 min-HPLC fraction of together c. 20 injections yielded c. 300 μg of lipophilic material, of which the 700 MHz NMR spectrum revealed the presence of long fatty acid chains (s. Fig. S2) though a direct identification through NMR was not possible due to low amounts.

The then following high-resolution mass spectrometry analyses allowed the identification of the active component as the well-known 1-oleoyl-*sn*-glycero-3-phosphocholine (lysophosphatidylcholine, LPC) together with *ca* 10% of the closely related and also active 1-stearoyl-*sn*-glycero-3-phosphocholine (see electronic supplementary material, Figs S3A, S4A and S5).

### Intrinsic production of LPC in honeybees and its presence in bumble bees and wasps

Activity against *P. larvae* was previously demonstrated in the isolated midgut tissue and peritrophic membrane of honeybees^24^. To authenticate the intrinsic production of LPC, the inhibitory growth effect against *P. larvae* from midguts of freshly emerged honeybees that emerged in their hives and honeybees artificially reared in our laboratory were compared (Table 1). Artificially reared larvae were obtained by grafting freshly laid eggs from combs taken from the outdoor hive into queen cells in a 48 multiwell plate and subsequent feeding and incubating under specified conditions (see materials). Gut extracts of bees artificially reared were as potent against *P. larvae* as those from midguts of naturally raised honeybees. A subsequent rearing experiment performed under axenic conditions using a larval diet containing penicillin G and streptomycin was carried out to rule out the possibility that the bacterial midgut microbiota might be responsible for the anti-*P. larvae* activity of honeybee midgut. The midguts of such microbe-free artificially reared honeybees possessed the same anti-*P. larvae* activity and their LPC content was in the same range as found for naturally raised imagines. Likewise, the influence of freshly emerged bees’ contact with nurse bees after their emergence was ruled out by testing the midgut extract of naturally raised bees that were denied contact to nurse bees. Also in this experiment, no noticeable reduction in midgut potency was found (Table 1).

Further, an ethanolic ablution of adult honeybees neither showed antibiotic activity against *P. larvae* nor contained lysophosphatidylcholine within the limits of LC-MS detection.

To widen our knowledge about LPC content in insects, midgut homogenates of *Vespa* sp. and *Bombus lapidarius* were also investigated despite the fact that these insects cannot be compared with honeybees with regard to their social habits and overall way of living. Both *Vespa* sp. and *B. lapidarius* showed an inhibitory activity against *P. larvae* in liquid medium. The presence of LPC in these guts was demonstrated through LC-MS, albeit its quantification was beyond of the scope of this investigation (see electronic supplementary material, Figs S3B,C, S4B,C and S5.)

### The antibacterial activity and potential spore-germination inhibiting properties of LPC and structurally related compounds against *P. larvae* and *M. plutonius*

In preliminary experiments, common antibacterial fatty acids such as oleic and linoleic acid could not be identified in the active fraction of midgut extract (data not shown). In royal jelly, our experiments showed that both 1-oleoyl-*sn*-glycero-3-phosphocholine and 1-stearoyl-*sn*-glycero-3-phosphocholine were detectable in traces, but outside the range of the calibration curve, i.e., below the limit of quantification, which means present at estimated concentrations lower than 0.3 ng/mg.

Once midgut homogenate activity against *P. larvae* had been demonstrated, we included *M. plutonius*, responsible for EFB, in our investigations. Given to-date available information on the antimicrobial activity of lipids, we further assessed the inhibitory activity of LPCs of different chemical structure and different origin together with a variety of lipidic compounds that share structural features with LPC against *P. larvae* and *M. plutonius*. The selection included phospholipid constituting lipids as well as different phospholipids, free fatty acids and two synthetically obtained phospholipid analogues, miltefosine and perifosine, which share key structural features with phospholipids (Table 2).

Remarkably, despite the variation in MICs calculated for unsaturated free fatty acid *vs* saturated free fatty acids, the activities of 1-oleoyl-*sn*-glycero-3-phosphocholine and 1-stearoyl-*sn*-glycero-3-phosphocholine were highly similar. These findings indicate that the growth inhibitory mechanism does not rely on the release of the antimicrobial free oleic acid as one could hypothesize based on the activity data for this fatty acid.

In an additional step, the spore-germinating and proliferation-inhibiting potential against *P. larvae* was assessed using LPC and miltefosine. A significant reduction in the number of CFU in both LPC- and miltefosine-containing samples (concentrations ≥5 μg/mL) as compared to the control was observed (Table 3).

### Assessment of the toxicity of LPC and structurally related compounds towards larvae

**Figure 1:**
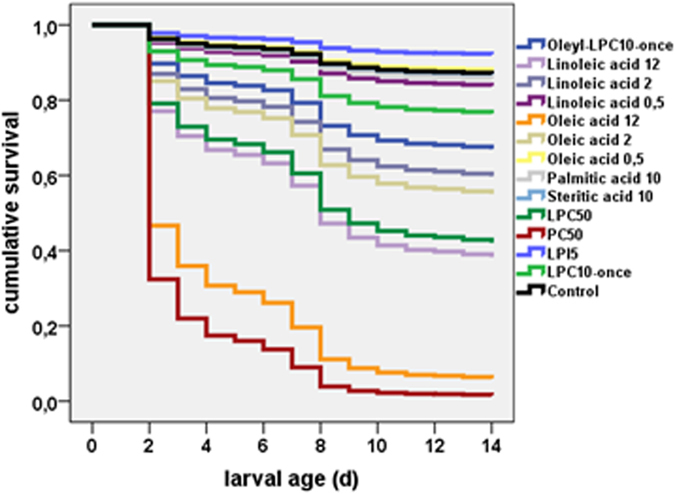
Cumulative survival rate of honeybee larvae fed with different doses (µg/larvae/d) of test compounds on day 1. The observation period was 14 d. Replicates: oleoyl-LPC (48/48/48), linoleic acid 12 (48), 2 (48/48) and 0.5 (48/48), oleic acid 12 (48), 2 (48/48) and 0.5 (48/48), palmitic acid (48/48), stearic acid (48/48), LPC 50 (48), PC (48), LPI 5 (48/48), LPC 10 (48/48/48), control (48/48/48/48/48/48/48/48).

The susceptibility of honeybee larvae against a variety of lipidic substances was assessed in a larval rearing assay, i.e., in an *in vivo* assay under *in vitro* conditions (Fig. 1).

As for all experiments with artificially reared larvae, one rearing plate contained 48 larvae, i.e., each 16 larvae from three different bee colonies, and is referred to in this section as one replicate. A Cox regression analysis was performed to study effects of different substances on larval mortality (not shown). For free fatty acids, a toxic effect was observed if these contained double bonds, e.g., oleic or linoleic acid. LPC was well tolerated at a concentration of 10 μg/d. Miltefosine fed in a dose of 10 μg/d/larva on day 1, 2 and 3 turned out to be toxic to larvae and was not further used in experiments with larvae (data not shown).

### LPC exhibits an *in vivo* protective effect against AFB, albeit not against EFB

**Figure 2:**
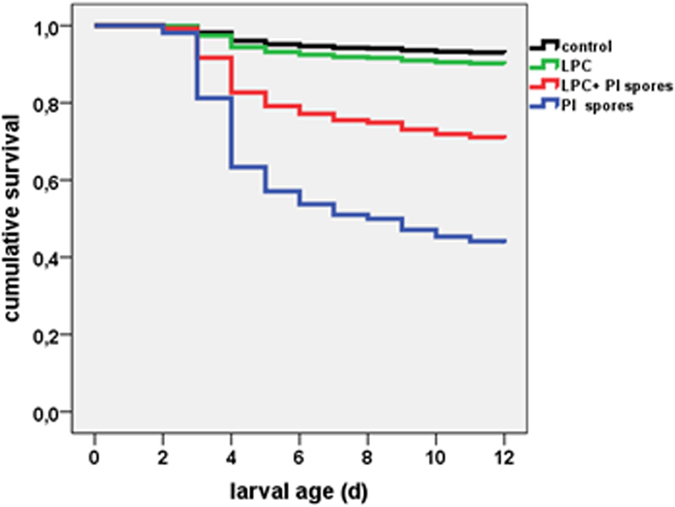
Cumulative survival rate of honeybee larvae. Control; fed with LPC (10 µg/d on day 1, 2 and 3); inoculated with *P. larvae* spores and treated with LPC (10 µg/d on day 1, 2 and 3) and larvae inoculated with *P. larvae* spores without treatment. The observation period was 12 d. Replicates: control (48/48/48/48/48/47). LPC (70/48/48/47/40), LPC + spores (71/48/48), spores (71/48/48).

These experiments were done to evaluate a potential usefulness of LPC in the combat of AFB or EFB. In a controlled exposure assay, larvae were artificially infected with a dose of ~50 *P. larvae* spores at day 1 and treated with LPC (10 μg/d/larva on day 1, 2 and 3) to assess its protective effect. LPC led to a significant reduction in larval mortality (Fig. 2, Table S1), i.e., LPC treatment could protect larvae from AFB.

**Figure 3:**
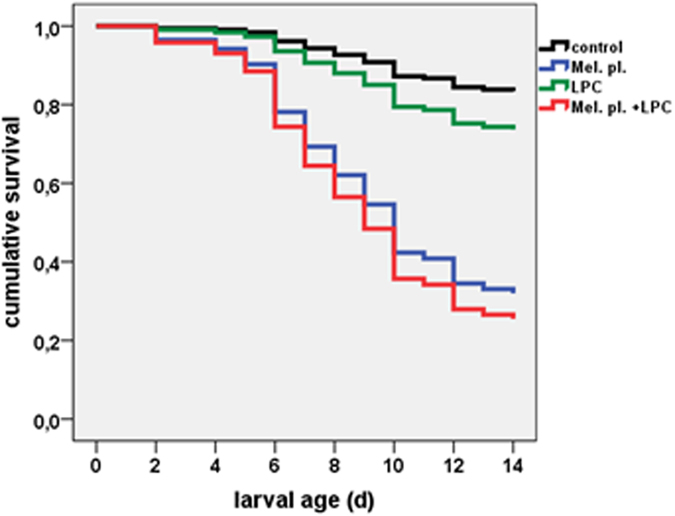
Cumulative survival rate of honeybee larvae. Control; fed with LPC (15 µg/d on day 1, 2 and 3); inoculated with *M. plutonius* bacteria and treated with LPC (15 µg/larva on day 1, 2, and 3) and larvae inoculated with *M. plutonius* without treatment. The observation period was 14 d; 2 replicates. Replicates: control (48/46), LPC (48/47), LPC + *M. plutonius* (48/48), *M. plutonius* (48/48).

In a second series of experiments, larvae were infected using *M. plutonius*-containing food at day 1. Surprisingly, LPC showed no enhancement of larval survival when applied even at doses of 15 μg/d for the first three days (Fig. 3, and Table S2). In a further experiment, the same dose of LPC was applied for 6 consecutive days obtaining the same negative result (data not shown). We have, however, not aimed at optimizing LPC doses and feeding regimes since this was not in the scope of the present investigation. From further rearing experiments, we observed that larvae tolerated 15 μg of LPC per larvae for six consecutive days.

### LPC is not toxic to adult honeybees at therapeutic doses

**Figure 4:**
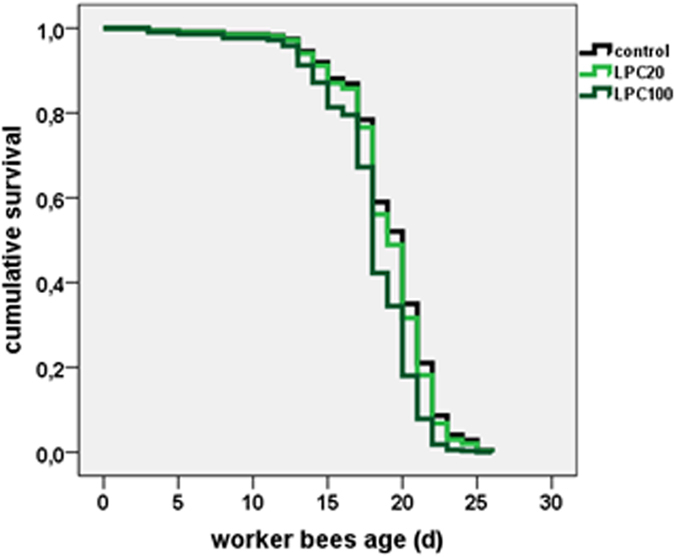
Cumulative survival rate of adult honeybee. Control; fed with LPC (20 and 100 µg/d up to day 26). *N* = 30 honeybees in each group.

The finding that LPC contributes to honeybee larval resistance against *P. larvae* opens the possibility to potential treatments using selected lipids, LPC or LPC-like compounds. We therefore at first assessed the susceptibility of adult honeybees against large doses of LPC by feeding 20 and 100 μg LPC/d, respectively, over a period of 26 d (Fig. 4). 20 μg/d were considered a realistic dose for a hive treatment in view of an application route from a nurse bee to larvae. The results indicate that LPC at 20 μg/d has no significant influence on the survival rate, whereas at the very high and biologically irrelevant dose of 100 μg/d, it leads to a very slightly increased mortality between day 15 and 22 (Table 4).

### LPC distribution pathways in a colony

**Figure 5:**
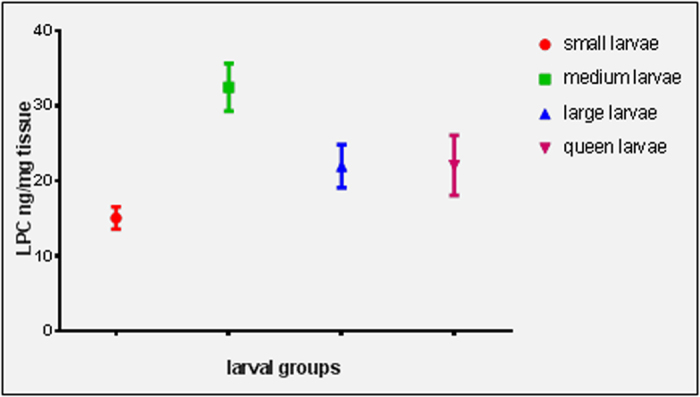
LPC content corresponding to fluorescence counts in larvae of different ages. Fluorescence values are normalized according to the background fluorescence in larvae.

To simulate a realistic application scenario, we fed fluorescent-labelled LPC in sugar solution to a starter colony. Fluorescence was detected in larvae of all three size groups (small, medium and large larvae) relating to age groups of 1.5–2, 2.5–3 and 5 d, respectively and corresponding to an average weight of *ca* 0.23–1 mg, *ca* 10–16 mg, and *ca* 45 mg, respectively. Fluorescence counts were normalized based on background fluorescence of untreated larvae and correspond to a respective LPC concentration and this concentration (>15 μg g^−1^) appears realistic in the prevention of AFB (see Fig. 5 and Table 5).

An ANOVA test was conducted to assess whether significant differences toward the LPC content exist within the different larval groups. Statistically significant differences in LPC content between larval groups compared to their controls were found (*F* = 20.884, *p* < 0.001). A Tukey post-hoc comparison revealed significant differences between each larval group and its corresponding control (*p*-value < 0.001). Results of the statistical analysis are given in Table S2 (electronic supplemental material).

## Discussion

LPC was identified as a constitutive antibacterial compound in honeybee midguts in both naturally developed and artificially reared honeybees. However, midgut activity decreased slightly when adult bees were fed on a protein and lipid free diet^23^. In the case of the latter, its origin from dietary sources, i.e., from food uptake or *via* contact to nurse bees, could be ruled out through feeding with almost LPC-free diet during artificial larval rearing. Hence, midgut homogenates and LPC levels in naturally developed bees and artificially reared bees showed equal anti-*P. larvae* activity. Lipids in general provide nutritional value and antimicrobial protection. Short- and long-chain fatty acids, the latter with increasing number of double bonds, show activity against *P. larvae*^27^. In a case study, palmitic, stearic, oleic, linoleic, and linolenic acid were found in 577 pollen samples investigated by Manning^28^. Consequently, free fatty acids, e.g., linoleic acid, in royal jelly and pollen are considered to help prevent bacterial infections in honeybee colonies through their antimicrobial properties^29^. The growth inhibitory effects of bee bread and pollen pellets against *P. larvae* were demonstrated by Crailsheim and Riessberger-Gallé^24^. Interestingly, Rinderer *et al*.^30^ observed that feeding pollen to 6–18 h old larvae reduced their susceptibility to *P. larvae* infections though not being able to prevent the outbreak of AFB. However, all naturally occurring dietary lipids do not provide AFB protection as found under LPC supplementation demonstrated through our experiments.

LPC is an intrinsically produced biomolecule, however, the further corroboration of its anti-*P. larvae* function through artificial blocking of all metabolic pathways that lead to LPC formation is not feasible. It is an amphiphilic phospholipid that occurs in various tissues and body fluids. LPC is present in vertebrates at physiologically low concentrations and is produced from phosphocholine through the activity of phospholipase enzymes, especially upon inflammatory stimuli. To date, little is known about phospholipid metabolism, turnover and homeostasis in insect midguts. Turunen and Kastari^31^ report on the metabolic fate of phosphatidylcholine in the gut of larvae of *Pieris brassicae* and revealed that dietary lecithin is converted to lysolecithin in the intestinal lumen, which is subsequently absorbed from the lumen and converted to phosphatidylcholine. However, no reports are so far available for honeybees and Apidae in general.

In a larval rearing assay, we could clearly demonstrate the protective effect of LPC-supplementation against AFB outbreak. The given dose resulted in a significantly reduced mortality by 30% in our chosen experimental setup with a mortality adjusted to 60% for the untreated group. However, the full anti-AFB potential of an LPC treatment at e.g. higher doses or in combination with other compounds was beyond the scope of our study.

Miltefosine, i.e., hexadecylphosphocholine, a structural analogue of alkyllysophospholipids showed an *in vitro* MIC of 2.5 μg/mL against *P. larvae*. Miltefosine is known for its antimicrobial activity, among other activities, toward pathogenic *Streptococcus* spp., against which it exerts a lytic effect^32^. However, in the present case, an *in vivo* AFB protective effect of miltefosine could not be demonstrated.

After the assessment of LPC’s *in vitro* anti-*P. larvae* activity and the proof of its *P. larvae* infection-preventing effect *in vivo* in a larval rearing assay, we aimed to emulate a most natural scenario of LPC delivery in a bee colony. Because of legal restrictions, infection trials with *P. larvae* in free-flying colonies are not possible. Application was simulated using fluorescent-labelled LPC. The aim was to demonstrate the existence of a delivery route which would lead to an anti-*P. larvae* relevant dose of LPC in small larvae susceptible to *P. larvae*. After application of the labelled LPC, fluorescence counts were measured in larvae and royal jelly and related to a concentration of LPC. We would like to emphasize the somewhat preliminary character of this experiment whose interpretation may pose difficulties, because i) the rather large and more hydrophilic fluorophore may bias bioavailability and ii) we do not know the metabolic behaviour of a lysophosphatidyl-fluorophore-conjugate in a biological system.

Young larvae are fed predominantly with jelly originating from the head glands, i.e., mandibular and hypopharyngeal glands, of nurse bees^33^. Up to 20% of young larvae’s diet consists of sugars indicating that honey stomach content is admixed^34^,^35^. With increasing age, increasing proportions of honey stomach content and pollen are added to the diet. Our feeding experiments using fluorescent-labelled LPC indicate that LPC is delivered to young larvae. This finding would be in accordance with the delivery of regurgitated honey stomach content to larvae. A passage of native LPC or LPC-fluorophore conjugate through head glands of nurse bees and their delivery to larvae remains unlikely. The concentration of the fluorophore in royal jelly was found below the limit of quantification (data not shown), which most likely indicates that it does not survive in native state the head gland passage. On the other hand, fluorescence was found in middle-aged and older larvae to a higher extent than in young larvae, which corroborates that the delivery route *via* honey stomach becomes increasingly important with larval age. The detected fluorescence corresponds to a concentration of ~15 μg/mL LPC in small larvae, which is high enough to exhibit an anti-*P. larvae* effect when one assumes that a concentration close to the given MIC of 2–5 μg/mL prevents *P. larvae* infection (Table 2). For older larvae, which are generally fed more sugar containing diet^36^ the calculated LPC concentration is even higher. Taken the results from the LPC delivery scenario together, we postulate that LPC supplementation provides a secondary anti-*P. larvae* benefit since LPC killing vegetative forms of *P. larvae* prevents the bacterium’s sporulation and therefore interrupts the ongoing infection circle in a colony.

Intriguingly, in our experimental design, we could not observe a protective effect of LPC supplementation toward the outbreak of EFB in larvae.

By demonstrating the *in vitro P. larvae* growth-inhibitory effect of LPC, its innoxiousness at therapeutic doses against both adult honeybees and larvae, its *in vivo* protective effect against *P. larvae* infection and by outlining a realistic delivery route in a colony, the presented work may offer new perspectives for a treatment of AFB without the utilization of classic antibiotics, that eventually lead to the formation of resistance. Moreover, since the activity of LPC against the spore-forming Gram-positive *P. larvae* was demonstrated and because LPC is easily available from e.g. soybean or egg yolk, the observed antimicrobial effect of LPC could be applied to other pathogenic bacteria, not only honeybee pathogens.

Altogether, these findings provide evidence of a yet undescribed defence mechanism against AFB in honeybees. LPC is present in honeybee midguts and coexisting with the intrinsic gut microbiota^6^, this points to a novel role of phospholipids in immune defence. The specificity in the host-parasite interaction honeybee-*P. larvae* may have co-evolved so that LPC release in the midgut of adult honeybees comprises a constitutive immune trait permanently active representing a first line of defence against AFB, whereas in aged larvae, tissue maturation (establishment of physical barriers) and potentially LPC levels both contribute to resistance. From the side of the parasite, which has co-evolved with *Apis mellifera*, there remains a window of approximately 48 h that can be exploited for its own benefit.

## Methods

### Bees, *Paenibacillus larvae* and used chemicals

For all experiments, *Apis mellifera carnica* was used. Bees and larvae were taken from regularly kept full sized colonies from the apiary located in the garden of the institute of zoology.

*P. larvae* ERIC II (SLU-233/00) was obtained from E. Forsgren (Uppsala, Sweden). We chose to work with ERIC II due to its high virulence at the individual level. The *P. larvae*-inhibitory potency of midgut against several field strains has been previously proven (Crailsheim & Riessberger-Gallé 2001). *Melissococcus plutonius* (strain 119) was obtained from Agroscope (Bern, Switzerland).

Phospholipids, miltefosine, perifosine and fatty acid reference compounds were obtained from Sigma-Aldrich (Wien, Austria). LPC used for the measurement of survival curves in adult honeybees was kindly provided by M. Hintersteiner (Bioseutica, Lugano, Switzerland). 17:0 LPC (1-*O*-heptadecanoyl-2-hydroxy-*sn*-glycero-3-phosphocholine) from Avanti Polar Lipids was used as an analytical standard (Alabaster, AL, USA).

### Identification and quantification of LPC in honeybee midgut

#### Preparation of honeybee midgut extracts

Bees were immobilized, sacrificed, and midguts were removed manually and prepared according to the following protocol: 10 guts were washed with insect ringer solution^24^, patted dry, and homogenized in 200 μL of water and 350 μL of ethanol (96%). For homogenization, ultrasound was applied for 3 s and the homogenate was stored 16 h overnight at 4 °C. Then the homogenate was centrifuged for 5 min (3000 rpm) and the pellet discarded. The supernatant was transferred to sterile Eppendorf vials and lyophilized. 10 midguts yielded ~5 mg of dried (lyophilized) midgut preparation, which was stored at 4–6 °C until it was utilized. Midgut preparations from bumble bees and wasps were obtained accordingly. The conditions of the bioactivity-guided fractionation of midgut extracts and the UPLC-HRMS method for identification of LPC^37^,^38^ can be found in the supplemental materials.

### Intrinsic production of LPC in honeybees

Anti-*P. larvae* activity of midgut extracts as assessed by growth inhibition in liquid medium was compared between midguts of naturally reared bees just before they emerged (cells were opened and fully developed bees were taken out for analysis) and midguts of naturally reared bees after they emerged and had contact to other bees. To confirm the intrinsic formation of anti-*P. larvae* activity in path-finding experiments, one group of larvae was reared under aseptic conditions adding penicillin G (0.01%) and streptomycin (0.01%) in the first larval diet and a second group was reared without antimicrobials.

To determine whether the substance originated from secretory glands or cells, and/or was present on the body surface of adult bees and then ingested, e.g., through a honeybee’s cleaning behaviour, 10 worker honeybees were rinsed with each 0.5 mL of ethanol (abs.). The combined ablutions were reduced to 20 μL and both tested for anti-*P. larvae* activity and analysed by HPLC using the same method as for the isolation of LPC.

### Assessment of antimicrobial activity against *P. larvae* and *M. plutonius* and *P. larvae* spore-inhibitory activity

Antibacterial assays were carried out using standard protocols to measure the turbidity caused by bacterial growth photometrically^39^. For *M. plutonius*, anaerobic conditions were applied^40^. For assessing the spore-inhibitory activity of LPC, spores and LPC were incubated in 1 mL of brain-heart infusion (BHI) and – in case of inhibitory activity – after plating the clear medium onto agar plates and further incubation, colony forming units (CFU) were counted. The presence of *P. larvae* in samples was confirmed using PCR as described in (Dobbelaere *et al*.)^41^. For further details, see supplemental materials.

### Artificial larval rearing protocol and infection procedure

A method modified from that reported by Aupinel *et al*.^42^ was used^36^. For infection trials with AFB, ~50 *P. larvae* spores/larva were added to the first diet. For trials with EFB, ~60,000 CFU/larvae were given. Larval diets containing infectious pathogens were added after the grafting, making up a total of 10 μL of larval diet on the first rearing day. These infectious doses resulted in a mortality of approximately 50% of larvae. One rearing plate contained 48 larvae, originating from each of three different bee colonies. Further details are given in supplemental materials.

### Survival of adult bees fed with LPC

Freshly emerged honeybees from three colonies were introduced separately in plastic cages in an incubator at 34.5 °C and 60% humidity, each cage containing 30 bees from one colony. A total of nine cages were used. Three cages contained 50% sucrose solution *ad libitum* and served as controls, each three cages had added 20 or 100 μg LPC per 30 μL sugar solution, respectively.

Initial experiments allowed us to assess the daily consumption of a caged honeybee to be 30 μL of sugar solution. The amount of consumption was again corroborated for all cages during this experiment (data not shown). Bees were kept in the incubator as long as they lived and mortality was recorded daily. Solutions were renewed every week to avoid fungal growth.

### Tracking of fluorescent-labelled LPC in a colony

A small starter colony kept in a polystyrene chamber composed of four combs and 1000–2000 individuals was used. Three combs were selected so that each of them contained either open honey cells, open brood cells with larvae of all ages and capped brood cells. A fourth comb contained open cells of young queen larvae. The colony was placed in the garden of the University of Graz and allowed to have a regular activity. A small recipient with 20 mL of sugar solution containing 20 mg LPC (0.1%) spiked with 400 μg of fluorescent-labelled LPC (2.0% in relation to the native LPC) from Atto-Tec Company (Siegen, Germany) was introduced into the colony. 20 h later bees had consumed all of the solution. Samples of larvae of different ages (1.5–2 d, 2.5–3 d and 5 d) were taken to measure fluorescence counts using a microplate reader (SpectraMax M3, Molecular Devices, Sunnyvale, CA, USA), with excitation/detection at λ = 480/525, respectively. Fluorescence values were normalized according to the background fluorescence in larvae.

## Additional Information

**How to cite this article**: Riessberger-Gallé, U. *et al*. Lysophosphatidylcholine acts in the constitutive immune defence against American foulbrood in adult honeybees. *Sci. Rep.* **6**, 30699; doi: 10.1038/srep30699 (2016).

## Supplementary Material

Supplementary Information

## Figures and Tables

**Table 1:** Summary of path-finding experiments with regard to the anti-*P. larvae* activity of midgut extracts of adult bees of different origin.

midgut origin	OE mean	SD	*N*
control (*P. larvae* growth in liquid medium)	0.59	0.049	15
artificially reared before emerging (no contact to others)	0.04	0.055	32
artificially reared after emerging (contact to others)	0.02	0.023	31
PenG/strep-reared before emerging (no contact to others)	0.08	0.044	19
PenG/strep-reared after emerging (contact to others)	0.01	0.014	48
hive bees before emerging (no contact to others)	0.00	0.003	51
*Vespa* sp.	0.00	0.005	5
*Bombus lapidarius*	0.00	0.003	5

Optical extinction (OE) is given as mean. (SD) = standard deviation, /*N* = number or replicates.

**Table 2:** Antimicrobial activity of fatty acids, phospholipids and phospholipid analogues against vegetative forms of *Paenibacillus larvae* and *Melissococcus plutonius* (MIC in μg/mL).

compound	*Paenibacillus larvae* ERIC II		*Melissococcus plutonius* strain 119	
	MIC (μg/mL)	MIC (μM)	MIC (μg/mL)	MIC (μM)
linoleic acid*	0.5	1.8	1	3.6
oleic acid*	1	3.5	1	3.5
palmitic acid*	>20	>78	>15	>40
stearic acid*	>20	>70	>10	>35
1-oleoyl-sn-glycero-3-phosphocholine	2	3.8	2	>3.8
1-stearoyl-sn-glycero-3-phosphocholine	5	9.5	3	>5.7
lysophosphatidylcholine‡ (soy bean)	2	†	3	†
phosphatidylcholine (bovine brain)	>50	†	>50	†
l-α-lysophosphatidylinositol	>50	>80	>10	>15
miltefosine	2.5	6.1	2	4.9
perifosine	not tested	not tested	2	4.3

^*^Solubilized in DMSO, final DMSO concentration not exceeding 1% in bacteria broth, experiments run in triplicate.

^†^Molecular weight not precisely defined due to mixtures containing different fatty acid chains.

^‡^LPC shows similar MIC values against ERIC I (30/06).

**Table 3:** Number of colony forming units (CFU) of *Paenibacillus larvae* after incubation of spores for 4, 6, and 8 d, respectively, with LPC and miltefosine.

tested compound	conc.(μg/mL)	number of CFU of *P. larvae*		
		mean (SD) 4 d/*N*	mean (SD) 6 d/*N*	mean (SD) 8 d/*N*
LPC*	2	no inhibition	—	—
LPC^*^	5	13.2 (2.5)/5	5.6 (2.8)/10	7.8 (3.3)/9
LPC*	10	11.0 (2.0)/5	7.8 (1.6)/5	6.0 (2.4)/10
miltefosine	2	no inhibition	—	—
miltefosine	5	7.8 (2.7)/5	5.0 (4.0)/11	6.9 (2.4)/10
miltefosine	10	13.6 (6.1)/5	8.6 (2.9)/5	8.3 (3.2)/10
control (+) Pen G	2	0	0	0
control (−) plated immediately	—	114.6 (28.4)/12	—	—

(SD) = standard deviation, /*N* = number or replicates.

^*^LPC from soybean.

**Table 4:** Results of the Cox regression analysis for mortality of adult bees fed with 20 or 100 μg/bee/day of LPC to assess toxicity.

	Wald	df	*p*-value	Exp(B)	95.0% CI for Exp(B)	
					Lower	Upper
control	10.189	2	0.006			
LPC 20	0.275	1	0.600	1.081	0.807	1.449
LPC 100	9.050	1	0.003	1.584	1.174	2.138

**Table 5:** Results of a Tukey post-hoc comparison.

(I) group (control)	(J) group (treated)	mean difference (J-I)	std. error	sig.	95% confidence interval	
					lower bound	upper bound
small larvae	small larvae	15.069	3.31503	0.002	−25.7143	−4.4238
medium larvae	medium larvae	32.466	4.21333	0.000	−45.9965	−18.9368
large larvae	large larvae	21.967	4.86514	0.002	−37.5896	−6.3438
queen larvae	3 d queen larvae	22.667	4.86514	0.002	−37.6896	−6.4438

Dependent variable: LPC ng/mg tissue Tukey HSD.
