# Supplementary material for: Lysophosphatidylcholine acts in the constitutive immune defence against American foulbrood in adult honeybees
Source: Sci Rep. 2016 Aug 2;6:30699. doi: 10.1038/srep30699 (PMC4969740; doi:10.1038/srep30699)
Supplement: Supplementary Information [file srep30699-s1.doc]

**Lysophosphatidylcholine acts in the constitutive immune defense against American foulbrood in adult honeybees**

Ulrike Riessberger-Gallé^1^, Javier Hernández-López^1^, Gerald Rechberger^2a,b^, Karl Crailsheim^1^, Wolfgang Schuehly^1,3,*^

^1^ Institute of Zoology, Universitätsplatz 2, Karl-Franzens-University Graz, 8010 Graz, Austria

^2a^ Institute of Molecular Biosciences, Karl-Franzens-University Graz, NAWI Graz, Humboldtstraße 50/II, 8010 Graz, Austria and ^2b^ OMICS Center Graz, BioTechMed-Graz

^3^ Institute of Pharmaceutical Sciences, Pharmacognosy, Universitätsplatz 4, Karl-Franzens-University Graz, 8010 Graz, Austria

* E-mail: wolfgang.schuehly@uni-graz.at (Wolfgang Schuehly).

**Electronic Supplementary Material:**

**Fig. S1.** HPLC run used in bioactivity-guided fractionation for the detection of anti-*P. larvae* activity. Fractions combining the eluate of each 5 min were combined and tested *in vitro* against *P. larvae*. Only the late eluting fractions (90-95 and 95-100 min, respectively) showed anti-*P. larvae* activity. Below the gradient (acetonitrile in water) is indicated.

**Fig. S2.** 700 MHz NMR spectrum of collected semi-preparative HPLC fractions 95-105 min of the yet unknown lipophilic substance, i.e. LPC. Spectrum taken in MeOD.


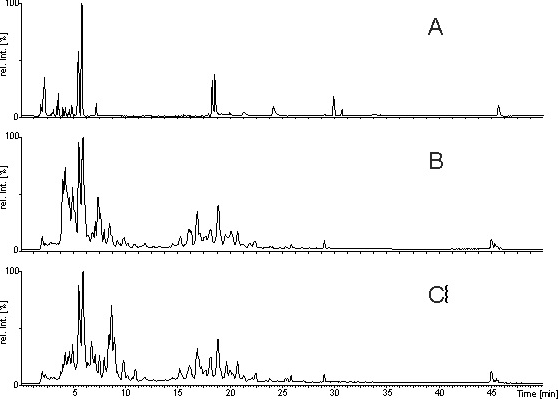


**Fig. S3.** LC-MS: Basepeak chromatograms of total lipid extract of honeybee midgut (A), bumble bee (B) and wasp (C).


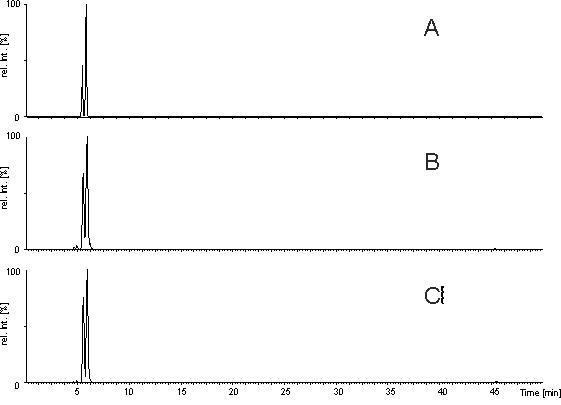


**Fig. S4.** LC-MS: Extracted ion chromatogram of *m/z* 522.3559 corresponding to the [M+H]^+^ ion of LPC 18:1 of the total lipid extract of honeybee midgut (A), bumble bee (B) and wasp (C). The double peak is caused by the structural isomers.


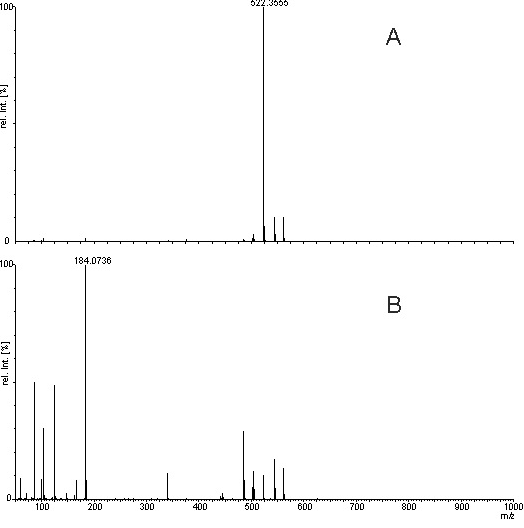


**Fig. S5.** LC-MS: Mass spectrum of the peak at RT 6 min shows the base peak signal at *m/z* 522.3555, which deviates only 1 ppm from the exact mass of the [M+H]^+^ ion of LPC 18:1 with *m/z* 522.3559. B: MS spectrum at RT 6 min shows a prominent fragment ion at *m/z* 184, indicating the presence of a phosphorylcholine headgroup.

***Bioactivity-guided fractionation of midgut extracts***

10 lyophilized midguts were prepared as above, and the lyophilized extract was dissolved in methanol-water (1:1) using ultrasound. Solid phase extraction (SPE cartridge, Isolute®, Biotage, Sweden; 100 µg, RP-18 (EC)) was carried out, eluting with a stepwise gradient of methanol in water (6:4, 7:3, 8:2, 9:1, and 100% methanol). The combined methanol-water (9:1) and pure methanol (100%) phases were dried *in vacuo*, and the residue was dissolved in methanol to yield a sample for further analysis and purification using HPLC.

Semi-preparative HPLC was carried out using an Agilent 1100 series (Agilent Technologies) instrument equipped with diode array detection (DAD). HPLC conditions were as follows: column: Knauer (Berlin) 125 x 4.6 mm, equipped with guard cartridge 4 x 4.6 mm, stationary phase Eurospher C18 (1.8 µm). The gradient elution program (flow rate 750 µl/min) was as follows: %acetonitrile in water (both with 0.1% TFA) (0→50 min/0→50%, 50→60 min/50→70%, 60→68 min/70→100%, 68→100 min/100%). HPLC conditions (elution and gradient program) were chosen initially due to their suitability for the fractionation of a protein- or peptide-containing extract, but these conditions were also found to be suitable for an extract containing lipidic compounds. In each run, the combined methanolic phases of the equivalent of 10 midguts (pre-purified on SPE, see above) were injected and the eluate was collected in fractions by time (5 min each). Each of the 20 fractions was subsequently lyophilized and tested *in vitro* against *P. larvae*, allowing the identification of two highly active fractions that eluted from 90–95 and 95–100 min. An aliquot of each of these active fractions was analysed using high resolution mass spectrometry (experimental conditions see below).

From 300 dried and lyophilised honeybee midguts (fresh weight 1650 mg, dry total weight 148 mg), 2.2 mg of a lipophilic compound was obtained (1.49% of dry weight corresponding to 1330 ng/mg fresh weight) and subjected to a preliminary proton NMR analysis. This analysis indicated the presence of long fatty acid chains, but no signals indicative of proteins and/or peptides were detected.

***Identification of LPC using high resolution mass spectrometry (UPLC-HRMS)***

Lipid analysis was performed using an AQUITY-UPLC system (Waters, Manchester, UK) equipped with a BEH-C18 column (2.1 x 150 mm, particle size 1.7 µm). The chromatographic conditions of the binary gradient were as follows: Solvent A consisted of water/methanol (1:1), solvent B was 2-propanol. Both solvents contained phosphoric acid (8 µM), ammonium acetate (10 mM) and formic acid (0.1%). The linear gradient started at 45% solvent B and increased to 100% solvent B within 32 minutes. The column compartment was kept at 50°C. A SYNAPT™G1 qTOF HD mass spectrometer (Waters) equipped with an ESI source was used for analysis. The following source parameters were used: capillary temperature 100 °C, desolvatization temperature: 400 °C, N_2_ as nebulizer gas. The capillary voltage was 2.6 kV in the positive and 2.1 kV in the negative ionization mode. Leucine-enkephaline ([M+H]^+^: *m/z* 556.2771 and [M–H]^–^: *m/z* 554.2615) was used as reference substance in the lock-spray. For further details see Knittelfelder *et al*. 2014. Data acquisition was performed using the MassLynx 4.1 software (Waters), for lipid analysis *Lipid Data Analyser* software was used (Hartler *et al*. 2011).

**(a) Assessment of antimicrobial activity against *P. larvae* and *M. plutonius***

During preliminary investigations, the demonstration of antimicrobial effects in liquid media is more precise and reliable than the visualization on agar plates due to the more homogenous growth of *P. larvae* in liquid media. Overnight cultures of the vegetative forms of *P. larvae* were prepared in BHI (brain heart infusion, 37 g/l, Oxoid) liquid medium. An inoculum of 50 µl from these overnight cultures was added to 1.0 ml of BHI liquid medium for use in *in vitro* bacterial tests in the presence or absence (control) of respective concentrations of test compounds. Penicillin G was used as positive control. Test tubes containing test compounds and controls were incubated for 24 h at 34.5 °C. The turbidity of the liquid medium was measured photometrically (Hernández-López *et al*. 2014). Minimum inhibitory concentrations (MIC) were obtained by interpolation of experi­mentally found extinction values for each of the different compound concentrations tested.

*M. plutonius* (strain 119) was cultured under anaerobic conditions in liquid Basal Medium (BM) for 4 days at 35 °C and from here serial dilutions were carried out to estimate CFU/ml by plating onto BM agar plates (Forsgren *et al*. 2013). 5 d later CFU counts led to an estimated 3.5*10^6^ CFU/ml. For the antimicrobial assay, an inoculum of 50 µl of the liquid culture was added to 1 ml of BM liquid medium. Three replicates per substance were included in the experiment. 5 d after the experiment, the turbidity was measured at 600 nm.

**(b) Measurement of *P. larvae* spore-inhibition activity**

To examine the antibacterial activity of LPC and miltefosine against *P. larvae* spores, testing was conducted in liquid cultures using brain-heart infusion BHI. As controls for growth, 1 ml of BHI with ~115 added spores of *P. larvae* (*n* = 12) and BHI as blank control (*n* = 5) were incubated at 34.5 °C each for 4, 6 or 8 d. As a positive control, 0.5 µl of penicillin G (1 mg/ml) was added to 1 ml BHI inoculated with ~115 spores (*n* = 5). All samples were checked visually for turbidity at day 4. Samples exhibiting no noticeable turbidity were plated on MYPGD agar, incubated for 6 d at 34.5 °C, and the number of CFUs per plate was counted. No CFUs observed two days after plating was interpreted as to indicate the absence of vegetative forms at the time of plating. The presence of *P. larvae* in samples was confirmed using PCR as described in (Dobbelaere *et al*. 2001). The observation intervals at d 4, 6 and 8 were chosen because larval mortality due to *P. larvae* in our rearing experiments was found highest between day 4 and 8.

To statistically compare the number of CFUs observed in samples with different treatment conditions (negative control, LPC, miltefosine), the non-parametric Kruskal-Wallis one-way analysis of variance (ANOVA) test was performed. This test was selected because the examined groups were of unequal size and not assumed to be normally distributed. In addition, pair-wise comparisons were performed by means of the non-parametric Mann-Whitney *U* test to detect significant differences between the groups.

**(c) Artificial larval rearing protocol and infection procedure**

A method modified from that reported in Aupinel *et al*. (2005) was used (Crailsheim *et al*. 2013) Larval experiments were carried out in order to test for the toxicity of different lipidic compounds in larval development and to carry out infection experiments with *P. larvae* spores and *M. plutonius* bacteria. Briefly, first instar worker larvae (5–10 h old) were grafted in 5 µl of larval diet with or without test compounds and another 5 µl of larval diet with or without *P. larvae* spores or *M. plutonius* bacteria, respectively. For infection trials with AFB, ~50 *P. larvae* spores/larva were added to the first diet. For trials with EFB, ~ 60,000 CFU/larvae were given. Larval diets containing infectious pathogens were added after the grafting, making up a total of 10 µl of larval diet on the first rearing day. These infectious doses resulted in a mortality of approximately 50% of larvae. Larvae were incubated at 34.5 °C and 95% relative humidity, which was reduced to 80% on day 7. Each larva was checked for its health state under the binocular microscope prior to feeding. A larva was defined as healthy when it was shiny and plump and movements of the spiracles could be observed. Cups with dead larvae were removed from the plate to prevent microbial cross-contamination. The mortality was checked daily until the end of the trials at day 12 or 14, respectively. Mortality of untreated larvae was found to be ~10%. One rearing plate contained 48 larvae, originating from each of 3 different bee colonies.

References:

Aupinel, P., Fortini, D., Dufour, H., Tasei, J.N., Michaud, B., Odoux, J.F. & Pham-Delègue, M-H. (2005) Improvement of artificial feeding in a standard *in vitro* method for rearing *Apis mellifera* larvae. *Bulletin of Insectology*, **58**, 107–11.

Crailsheim, K., Brodschneider, R., Aupinel, P., Behrens, D., Genersch, E., Vollmann, J. & Riessberger-Gallé, U. (2013) Standard methods for artificial rearing of *Apis mellifera* larvae. In: Dietemann, V., Ellis, J.D. & Neumann, P. editors. The COLOSS BEEBOOK, Volume I: standard methods for *Apis mellifera* research. Cambrian printers; *Journal of Apiculture Research*, **52**:1, 1–16 (http://dx.doi.org/10.3896/IBRA.1.52.1.05)

Dobbelaere, W., De Graaf, D.C., Peters, J.E. & Jacobs, F.J. (2001) Development of a fast and reliable diagnostic method for American foulbrood disease (*Paenibacillus* *larvae* subsp. *larvae*) using a 16S rRNA gene based PCR. *Apidologie*, **32**, 363–370.

Forsgren, E., Budge, G.E., Charrière, J-D. & Hornitzky, M.A.Z. (2013) Standard methods for European foulbrood research. In: Dietemann, V., Ellis, J.D. & Neumann, P. editors. The COLOSS BEEBOOK: Volume II: Standard methods for Apis mellifera pest and pathogen research. Cambrian printers; *Journal of Apiculture Research*, **52**:1, 1–14 (http://dx.doi.org/10.3896/IBRA.1.52.1.12)

Hartler, J., Trötzmüller, M., Chitraju, C., Spener, F., Köfeler, H.C. & Thallinger, G.G. (2011) Lipid Data Analyzer: unattended identification and quantitation of lipids in LC-MS data. *Bioinformatics,* **27**, 572–577.

Hernández-López, J., Schuehly, W., Crailsheim, K. & Riessberger-Gallé U*.* 2014 *In vitro* growth inhibition by *Hypericum* extracts and isolated pure compounds of *Paenibacillus larvae*, a lethal disease affecting honeybees worldwide. *Chemistry & Biodiversity*, **11**, 695–708.

Knittelfelder, O.L., Weberhofer, B.P., Eichmann, T.O., Kohlwein, S.D. & Rechberger, G.N. (2014) A versatile ultra-high performance LC-MS method for lipid profiling. *Journal of Chromatography B.* **951–952**:119–128.

**Table S1.** Results of a Cox regression analysis for mortality of larvae fed with infectious diet and infectious diet containing LPC at a concentration of 10 µg/larva/d for the first three days to assess protective effect against *P. larvae* infections.

|  | Wald | df | *p*-value | relative risk | 95,0% CI relative risk | |
| --- | --- | --- | --- | --- | --- | --- |
|  |  |  |  |  | lower | upper |
| control | 111.23 | 3 | 0.000 |  |  |  |
| LPC | 1.304 | 1 | 0.253 | 1.429 | 0.774 | 2.637 |
| LPC +*P. larvae* spores | 26.011 | 1 | 0.000 | 4.727 | 2.602 | 8.587 |
| *P. larvae* spores | 69.998 | 1 | 0.000 | 11.328 | 6.415 | 20.003 |
| replicates | 3.469 | 5 | 0.628 |  |  |  |
| LPC+*P. larvae* spores vs. *Pl* spores | 23.990 | 1 | 0.000 | 2.324 | 1.658 | 3.256 |

**Table S2.** Results of the Cox regression analysis for mortality of larvae fed with infectious diet and infectious diet containing LPC at a concentration of 15 µg/larva/d for the first three days to assess a protective effect against *M. plutonius* infections.

|  | Wald | df | *p*-value | relative risk | 95.0% CI relative risk | |
| --- | --- | --- | --- | --- | --- | --- |
|  |  |  |  |  | Lower | Upper |
| control | 81.598 | 3 | 0.000 |  |  |  |
| LPC | 2.544 | 1 | 0.111 | 1.684 | 0.888 | 3.193 |
| *M. plutonius* + LPC | 50.007 | 1 | 0.000 | 7.528 | 4.302 | 13.173 |
| *M. plutonius* | 40.608 | 1 | 0.000 | 6.282 | 3.570 | 11.056 |
